# Systematic Review of Antiretroviral-Associated Lipodystrophy: Lipoatrophy, but Not Central Fat Gain, Is an Antiretroviral Adverse Drug Reaction

**DOI:** 10.1371/journal.pone.0063623

**Published:** 2013-05-28

**Authors:** Reneé de Waal, Karen Cohen, Gary Maartens

**Affiliations:** Division of Clinical Pharmacology, Department of Medicine, University of Cape Town, Cape Town, South Africa; Institut National de la Santé et de la Recherche Médicale, France

## Abstract

**Background:**

Lipoatrophy and/or central fat gain are observed frequently in patients on antiretroviral therapy (ART). Both are assumed to be antiretroviral adverse drug reactions.

**Methods:**

We conducted a systematic review to determine whether fat loss or gain was more common in HIV-infected patients on ART than in uninfected controls; was associated with specific antiretrovirals; and would reverse after switching antiretrovirals.

**Results:**

Twenty-seven studies met our inclusion criteria. One cohort study reported more lipoatrophy, less subcutaneous fat gain, but no difference in central fat gain in HIV-infected patients on ART than in controls. Randomised controlled trials (RCTs) showed more limb fat loss (or less fat gain) with the following regimens: stavudine (versus other nucleoside reverse transcriptase inhibitors (NRTIs)); efavirenz (versus protease inhibitors (PIs)); and NRTI-containing (versus NRTI-sparing). RCTs showed increased subcutaneous fat after switching to NRTI-sparing regimens or from stavudine/zidovudine to abacavir/tenofovir. There were no significant between-group differences in trunk and/or visceral fat gain in RCTs of various regimens, but results from efavirenz versus PI regimens were inconsistent. There was no significant between-group differences in central fat gain in RCTs switched to NRTI-sparing regimens, or from PI-containing regimens.

**Conclusions:**

There is clear evidence of a causal relationship between NRTIs (especially thymidine analogues) and lipoatrophy, with concomitant PIs possibly having an ameliorating effect or efavirenz causing additive toxicity. By contrast, central fat gain appears to be a consequence of treating HIV infection, because it is not different from controls, is not linked to any antiretroviral class, and doesn't improve on switching.

## Introduction

Fat redistribution, also called lipodystrophy, is frequently observed in patients on long term antiretroviral therapy (ART) [1]. Some patients develop subcutaneous fat loss, or lipoatrophy; others gain fat, particularly in the breasts, dorsocervical fat pads, and viscerally. Individuals with mixed phenotypes of fat loss and fat gain also occur commonly. Fat redistribution is also associated with metabolic abnormalities, notably dyslipidaemia and insulin resistance, which increase the risk of cardiovascular disease [2].

Lipoatrophy has been associated with exposure to thymidine analogue nucleoside reverse transcriptase inhibitors (NRTIs) [3]. Central fat gain is also assumed to be an adverse drug reaction [4]. However, there is evidence that visceral abdominal fat in HIV-infected patients on ART is not increased relative to healthy controls [5]. Untreated HIV infection eventually results in wasting, including loss of adipose tissue. Fat gain, which is widely prevalent in the general population and increases with age, may in part be the result of effective ART reversing fat loss due to HIV infection. It is important to determine whether lipodystrophy is an adverse drug reaction to avoid unnecessary drug substitutions which may result in risks of virologic failure, new toxicities, and undermining patient confidence if the lipodystrophy does not improve. Treatment adherence is compromised when patients believe they have lipodystrophy from antiretrovirals [6].

If fat loss and fat gain were adverse antiretroviral drug reactions they would occur more commonly in HIV-infected patients on ART than in HIV-uninfected controls. Second, fat loss and/or fat gain would be associated with specific antiretroviral drugs or drug classes. Third, fat loss and/or fat gain would reverse after switching the identified antiretroviral drugs. We conducted a systematic review to test those three assumptions.

## Methods

### Eligibility criteria

#### Types of studies

To answer the question ‘Does fat loss and/or fat gain occur more commonly in patients on ART than in HIV-uninfected controls?’ we included prospective cohort studies comparing HIV-infected patients with ART exposure to population controls either known or presumed to be HIV-uninfected. To answer the questions ‘Is fat loss and/or fat gain associated with specific antiretroviral drugs?’ we included randomised controlled trials comparing antiretroviral regimens. To answer the question ‘Is fat loss and/or fat gain reversed after switching antiretroviral drugs?’ we included studies where participants with virologic suppression were randomised to continue their current ART regimen or switch to an alternative regimen.

#### Participants

We included both ART-naïve and ART-experienced HIV-infected patients who were at least 12 years old. For the cohort studies we included control participants who were presumed to be HIV-uninfected. We excluded studies with fewer than 20 participants in any arm.

#### Interventions

We included studies that used any antiretroviral regimens, given for at least 24 weeks, with the exception of those containing hydroxyurea.

#### Outcome measures

We included studies with at least one objective measure of fat distribution done at baseline, and repeated at least once, at a minimum of 24 weeks after baseline. Objective methods of measuring fat distribution included: dual-energy x-ray absorptiometry (DEXA), computerized tomography (CT), or magnetic resonance imaging (MRI). We included measures done both as primary or secondary outcomes, and in the whole study population, or within a sub-study. Specific outcomes included:

To assess fat loss:

- Change from baseline in limb fat
- Change from baseline in subcutaneous adipose tissue (SAT)
- Proportion with ≥20% loss in limb fat
- Proportion with ≥20% loss in SAT

To assess fat gain:

- Change from baseline in trunk fat
- Change from baseline in visceral adipose tissue (VAT)
- Proportion with ≥20% gain in trunk fat
- Proportion with ≥20% gain in VAT.

### Search strategies

We searched two electronic journal databases, PubMed and EMBASE, for articles published between 1 January 1990 and 7 July 2011. We hand-searched electronic databases for the Conferences on Retroviruses and Opportunistic Infections and the International AIDS Society conferences, from 2001. There was no language restriction, provided that an English translation of the abstract was available. The PubMed search strategy terms were as follows:

HIV Infections[MeSH] OR HIV[MeSH] OR hiv[tw] OR hiv-1*[tw] OR hiv-2*[tw] OR hiv1[tw] OR hiv2[tw] OR hiv infect*[tw] OR human immunodeficiency virus[tw] OR human immunedeficiency virus[tw] OR human immuno-deficiency virus[tw] OR human immune-deficiency virus[tw] OR ((human immun*) AND (deficiency virus[tw])) OR acquired immunodeficiency syndrome[tw] OR acquired immunedeficiency syndrome[tw] OR acquired immuno-deficiency syndrome[tw] OR acquired immune-deficiency syndrome[tw] OR ((acquired immun*) AND (deficiency syndrome[tw])) OR “sexually transmitted diseases, viral”[MH:noexp]

AND

Search Antiretroviral Therapy, Highly Active[MeSH] OR Anti-Retroviral Agents[MeSH] OR Antiviral Agents[MeSH:NoExp] OR ((anti) AND (hiv[tw])) OR antiretroviral*[tw] OR ((anti) AND (retroviral*[tw])) OR HAART[tw] OR ((anti) AND (acquired immunodeficiency[tw])) OR ((anti) AND (acquired immunedeficiency[tw])) OR ((anti) AND (acquired immuno-deficiency[tw])) OR ((anti) AND (acquired immune-deficiency[tw])) OR ((anti) AND (acquired immun*) AND (deficiency[tw]))

OR

Search zalcitabine OR zidovudine OR lamivudine OR stavudine OR didanosine OR tenofovir OR abacavir OR emtricitabine OR nevirapine OR efavirenz OR delavirdine OR etravirine OR rilpivirine OR amprenavir OR atazanavir OR tipranavir OR indinavir OR saquinavir OR lopinavir OR fosamprenavir OR ritonavir OR darunavir OR nelfinavir OR enfurvirtide OR maraviroc OR raltegravir

AND

Search Lipodystrophy[mh] OR lipodystrophy[tiab] OR lypodystrophy[tiab] OR lipodystrophies[tiab] OR lipohypertrophy[tiab] OR lipoatrophy[tiab] OR body fat distribution[mh] OR fat[tiab] OR fats[tiab] OR abdominal fat[mh] OR adipose tissue[mh] OR adipose [tiab] OR adiposity[tiab] OR temporal wasting[tiab] OR buffalo hump[tiab].

### Data collection

Two authors (RdW and KC) independently reviewed all study abstracts identified by the search strategy, using a specially designed eligibility form. We obtained the full articles, conference abstracts or conference posters for all studies that met the inclusion criteria. An independent translator reviewed articles that were published in languages other than English. We resolved disagreements as to study eligibility through consensus and discussion with the third author (GM) if necessary. One author (RdW) extracted data using a data extraction form; another author (KC) checked the extracted data. Two authors (RdW and KC) assessed the risk of bias of all included studies [7].

## Results

**Figure 1:**
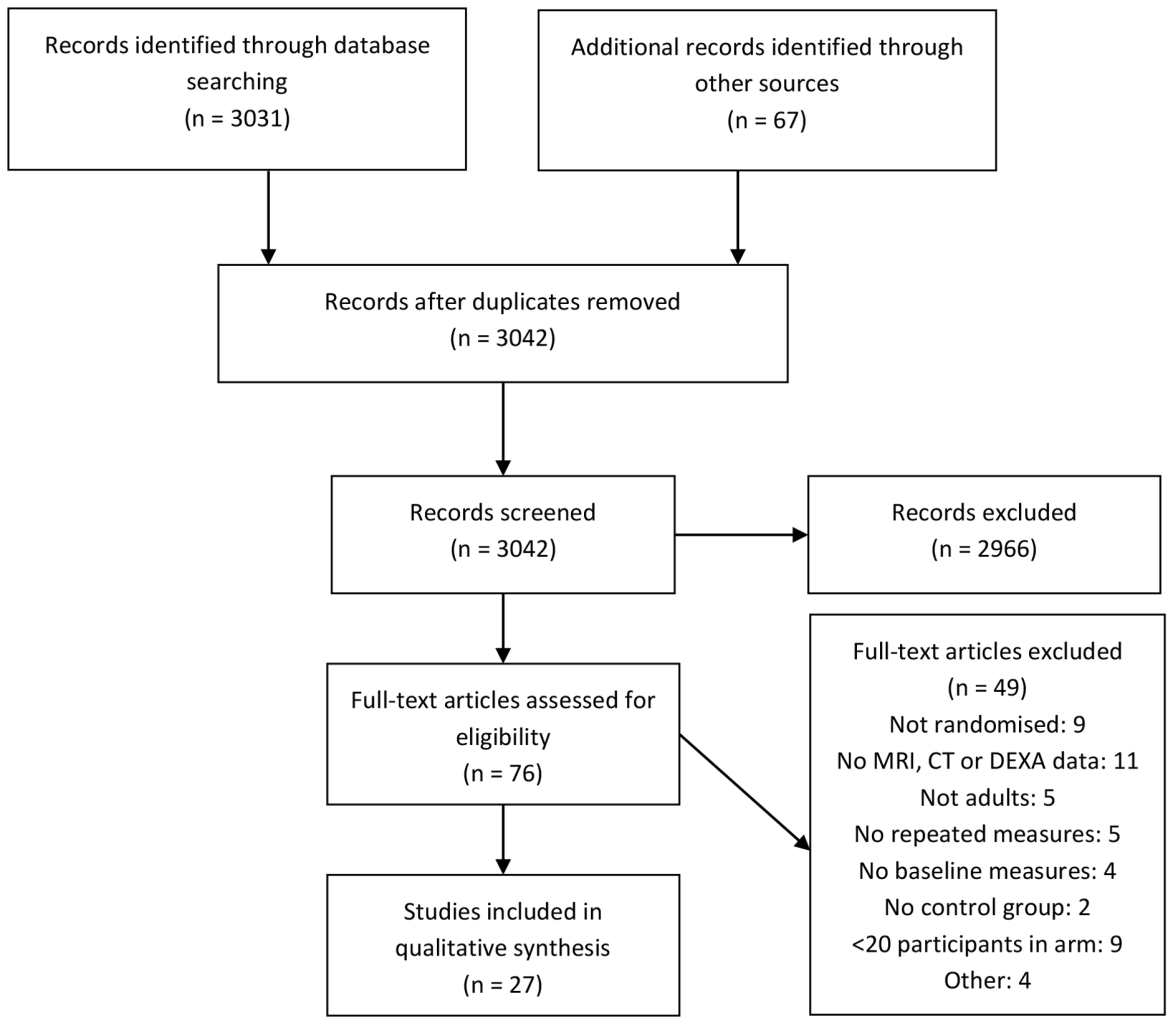
Flow diagram of study selection.

We identified a total of 27 studies for inclusion in the review: one cohort study comparing HIV-infected patients with controls [8]; 18 randomised controlled trials comparing antiretroviral regimens [3], [4], [9]–[24]; seven switching studies [25]–[31]; and one study that fulfilled the criteria for both randomised controlled trials and switching studies [32]. Our search of PubMed and Embase databases identified 3031 potential abstracts. We identified a further 67 articles through conference databases or other sources. After removing duplicate records, we screened 3042 abstracts and excluded 2966 as they did not meet our inclusion criteria. We retrieved and assessed the full text articles for the remaining 76, and excluded 49 that did not meet our inclusion criteria (see Figure 1 and Table S1). We assessed two articles that were published in Spanish through means of a translator. We included the full text articles for five studies that we identified through conference abstracts.

### Characteristics of included studies

Fat distribution measures were done in all participants for 15 studies: one cohort study [8]; nine randomised controlled trials comparing antiretroviral regimens [4], [12], [13], [15], [16], [18], [19], [23], [32]; and five switching studies [25], [27], [29]–[31]. They were done in a subset of participants only in the remaining 12 studies [3], [9]–[11], [14], [17], [21], [22], [24], [26], [28], [33]. The characteristics of the included studies are summarised in Table 1, and risk of bias assessment in Table S2.

**Table 1:** Characteristics of included studies.

Study	Year published	DEXA/CT/MRI population	n	ART experience	Lipodystrophy	Age group	Duration of follow up	Primary endpoint(s)
[8]	2010	Whole study	691	Experienced	Patients with and without lipodystrophy included	≥18 years	5 years	SAT, VAT
[3]	2011	Sub-study	269	Naïve	NA	≥16 years	192 weeks	Lipoatrophy (DEXA)
[9]	2011	Sub-study	63	Naïve	NA	NR	96 weeks	Efficacy
[10]	2010	Sub-study	112	Naïve	NA	≥18 years	96 weeks	Efficacy
[11]	2010	Sub-study	156	Experienced	NR	≥18 years	48 weeks	Efficacy
[4]	2009	Whole study	66	Experienced	NR	≥18 years	48 weeks	Mitochondrial changes
[12]	2009	Whole study	48	Naïve	NA	18–70	24 months	LF, SAT, VAT
[13]	2009	Whole study	757	Naïve	NA	≥13 years	96 weeks	Lipoatrophy (DEXA)
[14]	2009	Sub-study	47	Experienced	Patients with and without lipodystrophy included	Adult	48 weeks	Efficacy
[15]	2009	Whole study	200	Naïve	NA	Adult	96 weeks	Efficacy and safety
[32]	2009	Whole study	101	Experienced	Self-reported lipoatrophy	NR	48 weeks	STF
[16]	2009	Whole study	357	Experienced	NR	≥18 years	96 weeks	Efficacy
[17]	2008	Sub-study	140	Naïve	NA	≥18 years	48 weeks	LF
[18]	2008	Whole study	117	Naïve	NA	≥18 years	96 weeks	LF at 96 weeks
[19]	2008	Whole study	155	Naïve	NA	NR	96 weeks	Efficacy
[20], [33]	2007	Sub-study	157	Naïve	NA	NR	144 weeks	Changes in glucose and lipid metabolism
[21]	2007	Sub-study	62	Experienced	Patients with and without lipodystrophy included	NR	>96 weeks	LF
[22]	2007	Sub-study	57	Naïve	NA	Adult	96 weeks	Clinical lipoatrophy
[23]	2006	Whole study	105	Experienced	Lipoatrophy	≥18 years	48 weeks	LF
[24]	2006	Sub-study	211	Naïve	NA	≥16 years	48 weeks	VAT
[25]	2012	Whole study	200	Experienced	Abdominal fat accumulation	≥18 years	96 weeks	TF:LF
[26]	2012	Sub-study	74	Experienced	NR	≥18 years	48 weeks	VAT
[27]	2011	Whole study	142	Experienced	NR	Adult	48 weeks	LF
[28]	2009	Sub-study	100	Experienced	NR	≥18 years	48 weeks	Change in haemoglobin
[29]	2008	Whole study	100	Experienced	Self-reported lipoatrophy	≥18 years	96 weeks	STF
[30]	2002	Whole study	106	Experienced	Clinical lipoatrophy	>18 years	24 weeks	LF
[31]	2001	Whole study	106	Experienced	Clinical lipodystrophy	Adult	48 weeks	Efficacy

LF: limb fat; NA: not applicable – treatment-naïve patients; NR: not reported; SAT: subcutaneous adipose tissue; STF: subcutaneous thigh fat; TF: trunk fat; VAT: visceral adipose tissue.

### Participants

Twelve randomised controlled trials enrolled antiretroviral-naïve participants [3], [9], [10], [12], [13], [15], [17]–[19], [22], [24], [33]. The cohort study [8], seven randomised controlled trials [4], [11], [14], [16], [21], [23], [32], and all the switching studies enrolled antiretroviral-experienced participants. The cohort study enrolled participants both with and without lipodystrophy. Two randomised controlled trials enrolled only participants with clinical or self-reported features of lipoatrophy [23], [32]; and two enrolled participants both with and without lipoatrophy [14], [21]. Three switching studies enrolled only participants with clinical or self-reported lipoatrophy [29]–[31]; and one enrolled participants with features of abdominal fat accumulation, defined as a waist-to-hip ratio of >0.9, with a waist circumference >88.2 or >75.3 cm in men and women respectively [25]. For the remaining studies it was not reported whether or not participants had features of lipodystrophy at baseline.

### Interventions

All included studies involved a comparison of different antiretroviral regimens, with the exception of the cohort study that compared HIV-infected people with people who were known or presumed to be HIV-uninfected [8]. Eight randomised controlled trials [4], [9], [11]–[13], [18], [21], [32], and two switching studies [29], [32] evaluated NRTI-sparing regimens; four randomised controlled trials [3], [19], [24], [33], and one switching study [31] evaluated protease inhibitor (PI) versus non-nucleoside reverse transcriptase inhibitor (NNRTI) regimens; six randomised controlled trials [3], [14], [16], [22], [23], [33], and five switching studies [27], [28], [30], [32], [34] evaluated NRTI versus NRTI regimens; two randomised controlled trials [15], [17], and one switching study [25] evaluated PI versus PI regimens; and one randomised controlled trial [10], and one switching study [26] evaluated other antiretroviral categories.

### Outcomes

An objective measure of fat distribution was the primary study endpoint in 15 studies [3], [8], [12], [13], [17], [18], [21], [23]–[27], [29], [30], [32]. In the remaining studies, measures of fat distribution were secondary endpoints.

#### HIV-infected patients compared with healthy controls

The FRAM2 study compared fat distribution in HIV-infected patients with healthy controls, using MRI at two time-points separated by about five years [8]. The control participants were recruited from the Visceral Fat and Metabolic Rate in Young Adults sub-study of the Coronary Artery Risk Development in Young Adults study. They were selected as they had previous experience of fat distribution investigations, and had a similar age and ethnic distribution to most HIV-infected patients in the United States [35]. Although the analyses included HIV-infected patients who had never been on ART (11.8% at baseline and 5.7% at year 5), we included the study as only a small minority were not on ART. Sub-group analyses by ART status were not done.

There was clear evidence of fat loss in HIV-infected people, 53% of whom had lipoatrophy (defined as leg SAT below the 10^th^ percentile of controls) after five years of observation. Longer duration of stavudine use was associated with less leg SAT. After five years there was significantly less SAT at all sites in HIV-infected men, but only in the limbs in HIV-infected women. Multivariable analysis showed that increase in SAT over five years was less in HIV-infected people at all sites except the lower trunk.

By contrast, there was no evidence of regional fat accumulation in HIV-infected people. After five years the amount of trunk SAT and VAT was similar in HIV-infected and control women, while HIV-infected men had less fat at all sites than control men. The gains in VAT over five years were similar in HIV-infected people and controls.

#### Fat loss: changes in limb fat and SAT with different antiretroviral regimens

The changes from baseline in limb fat and SAT are summarised in Tables 2, 3, 4, 5, and 6, and the incidences of peripheral lipoatrophy (defined as ≥20% loss in limb fat) are summarised in Tables 7, 8, 9, 10, and 11.

**Table 2:** Change from baseline in limb fat (LF) on DEXA scan, and subcutaneous thigh fat (STF) and subcutaneous adipose tissue (SAT) on CT scan: NRTI-containing regimens versus NRTI-sparing regimens.

Study	Measure	Arm	Week 48 (n)	p value	Week 96 (n)	p value
[9]	LF^a^	LPVr+AZT+3TC	−703 g (22)	0.014	−1930 g (5)	>0.05
		LPVr monotherapy	−63 g (41)		−400 g (8)	
[4]			Percentage of body fat, 0 & 48 wks:^c^			
	LF^a,b^	LPVr+ continue 2NRTIs	12.2; 13.4 (33^d^)	NR	ND	NA
		NVP+LPVr	11.1; 14.1 (33^d^)		ND	
[12]			Mean (95% CI) g, 0 & 48 wks:^f^		Mean (95% CI) g, 96 wks:^f^	
	LF^e^	AZT+3TC+LPVr	6360 (5919 to 6801); 6520 (6059 to 6981) (22^d^)	NR	5980 (5519 to 6441)	NR
		NVP+LPVr	6360 (5968 to 6752); 7030 (6618 to 7442) (26^d^)		7210 (6789 to 7631)	
			Mean (95% CI) cm^2^, 0 & 48 wks:^f^		Mean (95% CI) cm^2^, 96 wks:^f^	
	SAT^e^	AZT+3TC+LPVr	118 (107 to 129); 126 (114 to 138) (22^d^)	NR	123 (111 to 135)	NR
		NVP+LPVr	118 (108 to 128); 132 (121 to 143) (26^d^)		142 (131 to 153)	
[13]	LF^a^	EFV+3TC+(TDF or AZT or D4T)	8.9%^g^ (188)	NR	1.4% (171)	<0.001 vs NRTI-sparing
		LPVr+3TC+(TDF or AZT or D4T)	10.1%^g^ (191)		9.8% (166)	0.013 vs NRTI-sparing
		EFV+LPVr (NRTI-sparing)	14.2%^g^ (197)		17.6% (173)	
[32]	STF^a^	ABC+continue other ARVs	18% (42)	0.57	ND	NA
		LPVr+NVP	17% (46)		ND	
	SAT^a^	ABC+continue other ARVs	29% (43)	0.6	ND	NA
		LPVr+NVP	33% (47)		ND	
[18]	LF^e^	PIr+2NRTIs	0.37 kg (28)	0.253 vs NNRTI+PIr	ND	NA
		NNRTI+2NRTIs	0.9 kg (21)	0.298^h^ vs PIr+2NRTIs	ND	NA
		NNRTI+PIr	0.79 kg (49)	0.793 vs NRTI+2NRTIs	ND	NA
[21]	LF^a^	EFV+2NRTIs^i^	−242 g (25)	0.086	−850 g (25)^j^	0.002
		LPVr+EFV	562 g (22)		782 g (22)^j^	
[11]	LF^a^	DRVr+ continue 2NRTIs	−0.26% (74)	<0.001	ND	NA
		DRVr monotherapy	8.3% (67)		ND	NA

a. median; b. leg fat; c. absolute values at each time-point (change from baseline not reported); d. n at baseline (n at time-point not reported); e. mean; f. means (corrected for differences in baseline values) and 95% confidence intervals (calculated by the authors of this review) at each time-point (change from baseline not reported); g. values derived from graph; h. calculated by authors of this review; i. DDI+3TC or DDI+AZT or AZT+3TC or D4T+ 3TC or DDI+D4T; j. at last visit (median 102 weeks).

NA: not applicable; ND: not done; NR: not reported.

Antiretrovirals: 3TC: lamivudine; ABC: abacavir; AZT: zidovudine; D4T: stavudine; DRVr: ritonavir-boosted darunavir; EFV: efavirenz; LPVr: ritonavir-boosted lopinavir; NVP: nevirapine; PIr: ritonavir-boosted PI; TDF: tenofovir.

**Table 3:** Change from baseline in limb fat (LF) on DEXA scan and subcutaneous adipose tissue (SAT) on CT scan: PI versus NNRTI.

Study	Measure	Arm	Week 48 (n)	p value	Week 96 (n)	p value
[3]	LF^a^	ATVr+(ABC+3TC or TDF+FTC)	25.2%^b^ (105)	NR	30.4% (94)	0.01
		EFV+(ABC+3TC or TDF+FTC)	17.7%^b^ (112)		16.5% (109)	
[13]	LF^c^	LPVr+3TC+(TDF or AZT or D4T)	10.1%^b^ (191)		9.8% (166)	0.007
		EFV+3TC+(TDF or AZT or D4T)	8.9%^b^ (188)	NR	1.4% (171)	
[18]	LF^a^	PIr+2NRTIs	0.37 kg (28)	0.30^d^	ND	NA
		NNRTI+2NRTIs	0.9 kg (21)		ND	NA
[19]	LF^c^	LPVr+AZT+3TC or LPV monotherapy^e^	11.8% (NR)	NR	18.5%^b^ (74)	NR
		EFV+AZT+3TC	3.1% (NR)	NR	−9%^b^ (32)	
[24]	SAT^a^	ATV+AZT+3TC	12.8 cm^2b^ (62). 95% CI for difference: −16.8 to 27.5	NR	ND	NA
		EFV+AZT+3TC	7.6 cm^2b^ (47)		ND	NA
[20], [33]	LF^c,f^	NFV+(AZT+3TC or DDI+D4T)	−4.7%^b^ (23)	NR	−23.7%^b^ (11)	NR
		EFV+(AZT+3TC or DDI+D4T)	1.5%^b^ (26)		9.9%^b^ (16)	

a. mean; b. values derived from graph; c. median; d. calculated by authors of this review; e. if virologically suppressed for 3 months; f. as-treated analysis.

NA: not applicable; ND: not done; NR: not reported.

Antiretrovirals: 3TC: lamivudine; ABC: abacavir; ATV: atazanavir; ATVr: ritonavir-boosted atazanavir; AZT: zidovudine; D4T: stavudine; DDI: didanosine; EFV: efavirenz; FTC: emtricitabine; LPVr: ritonavir-boosted lopinavir; NFV: nelfinavir; TDF: tenofovir.

**Table 4:** Change from baseline in limb fat (LF) on DEXA scan and subcutaneous adipose tissue (SAT) on CT scan: NRTI versus NRTI.

Study	Measure	Arm	Week 48 (n)	p value	Week 96 (n)	p value
[3]	LF^a^	ABC+3TC+(ATVr or EFV)	22.8%^b^ (107)	NR	24.9% (102)	0.46
		TDF+FTC+(ATVr or EFV)	19.7%^b^ (110)		20.9% (101)	
[14]	LF^c^	ABC+3TC+continue PI or NNRTI	104 g (23)	0.92	ND	NA
		TDF+FTC+continue PI or NNRTI	75 g (24)		ND	NA
[22]	LF^a^	ABC+3TC+EFV	686 g (25)	0.001	913 g (25)	<0.001
		D4T+3TC+EFV	−1164 g (32)		−1578 g (32)	
[23]	LF^a^	ABC+continue other ARVs	483 g (44)	0.37	ND	NA
		TDF+continue other ARVs	329 g (49)		ND	NA
	SAT^a^	ABC+continue other ARVs	8.0 cm^2^ (44)	0.96	ND	NA
		TDF+continue other ARVs	8.4 cm^2^ (49)		ND	NA
[20], [33]	LF^c,d^	DDI+D4T+(EFV or NFV)	−11.9%^b^ (42)	NR	−26.4%^b^ (22)	NR
		AZT+3TC+(EFV or NFV)	1.6%^b^ (39)		1.7%^b^ (24)	
[16]	LF^a^	ABC+3TC+continue NNRTI or PI	0.3 kg (179^e^)	0.4	0.53 kg (NR)	0.46
		TDF+FTC+continue NNRTI or PI	0.19 kg (178^e^)		0.42 kg (NR)	

a. mean; b. values derived from graph; c. median; d. as-treated analysis; e. n at baseline (n at time-point not reported).

NA: not applicable; ND: not done; NR: not reported.

Antiretrovirals: 3TC: lamivudine; ABC: abacavir; ATV: atazanavir; ATVr: ritonavir-boosted atazanavir; AZT: zidovudine; D4T: stavudine; DDI: didanosine; EFV: efavirenz; FTC: emtricitabine; NFV: nelfinavir; TDF: tenofovir.

**Table 5:** Change from baseline in limb fat (LF) on DEXA scan and subcutaneous adipose tissue (SAT) on CT scan: PI versus PI.

Study	Measure	Arm	Week 48 (n)	p value	Week 96 (n)	p value
[15]	LF^a^	ATVr+D4T+3TC	2% (72)	NR	−9% (55)	>0.05
		ATV+D4T+3TC	−3% (89)		−17% (67)	
	SAT^a^	ATVr+D4T+3TC	12% (68)	NR	8% (56)	>0.05
		ATV+D4T+3TC	12% (85)		2% (62)	
[17]	LF^b^	TPVr100+TDF+3TC	1.4% (46)	0.02 vs LPVr+TDF+3TC	ND	NA
		TPVr200+TDF+3TC	1.6% (48)	0.14 vs LPVr+TDF+3TC	ND	NA
		LPVr+TDF+3TC	2.8% (45)		ND	NA
	SAT^b^	TPVr100+TDF+3TC	−2.1 cm^2^ (46)	0.03 vs LPVr+TDF+3TC	ND	NA
		TPVr200+TDF+3TC	4.2 cm^2^ (48)	0.13 vs LPVr+TDF+3TC	ND	NA
		LPVr+TDF+3TC	17.6 cm^2^ (45)		ND	NA

a. mean; b. median.

NA: not applicable; ND: not done; NR: not reported.

Antiretrovirals: 3TC: lamivudine; ATV: atazanavir; ATVr: ritonavir-boosted atazanavir; D4T: stavudine; LPVr: ritonavir-boosted lopinavir; TDF: tenofovir; TPVr100: tipranavir/ritonavir 500/100 mg twice a day; TPVr200: tipranavir/ritonavir 500/200 mg twice a day.

**Table 6:** Change from baseline in limb fat (LF) on DEXA scan and subcutaneous adipose tissue (SAT) on CT scan: raltegravir versus efavirenz.

Study	Measure	Arm	Week 48 (n)	p value	Week 96 (n)	p value
[10]	LF^a^	RAL+TDF +FTC	18.1% (40)	0.95^b^	18.2% (37)	0.88^b^
		EFV+TDF +FTC	17.7% (46)		17.0% (38)	

a. mean; b. p value calculated by authors of this review.

Antiretrovirals: EFV: efavirenz; FTC: emtricitabine; RAL: raltegravir; TDF: tenofovir.

**Table 7:** Proportion of patients with peripheral lipoatrophy on DEXA scan: NRTI-containing regimens versus NRTI-sparing regimens.

Study	Definition	Arm	Week 48 (n)	p value	Week 96 (n)	p value
[9]	>20% loss of LF	LPVr+AZT+3TC	27.3% (22)	0.018	ND	NA
		LPVr monotherapy	4.9% (41)		ND	
			OR^a^ LPVr+AZT+3TC vs LPVr monotherapy 7.06. 95% CI 1.11 to 78.69			
[13]	≥20% loss of LF	EFV+3TC+(TDF or AZT or D4T)	21% (188)	NR	32% (171)	<0.001 vs NRTI-sparing
		LPVr+3TC+(TDF or AZT or D4T)	10% (191)		17% (166)	0.023 vs NRTI-sparing
		EFV+LPVr (NRTI-sparing)	7% (197)		9% (173)	
[11]	>20% loss of LF	DRVr+ continue 2NRTIs	10.8% (74)	0.035	ND	NA
		DRVr monotherapy	1.5% (67)		ND	NA

a. adjusted for age and sex.

LF: limb fat; NA: not applicable; ND: not done; NR: not reported; OR: odds ratio.

Antiretrovirals: 3TC: lamivudine; AZT: zidovudine; D4T: stavudine; DRVr: ritonavir-boosted darunavir; EFV: efavirenz; LPVr: ritonavir-boosted lopinavir; TDF: tenofovir.

**Table 8:** Proportion of patients with peripheral lipoatrophy on DEXA scan: PI versus NNRTI.

Study	Definition	Arm	Week 48 (n)	p value	Week 96 (n)	p value
[13]	≥20% loss of LF	LPVr+3TC+(TDF or AZT or D4T)	10% (191)	NR	17% (166)	0.003
		EFV+3TC+(TDF or AZT or D4T)	21% (188)		32% (171)	
					OR^a^ EFV vs LPVr 2.63. 95% CI 1.49 to 4.64	<0.001
[19]	>20% loss of LF	LPVr+AZT+3TC or LPVr monotherapy^b^	ND	NA	5% (74)	<0.001
		EFV+AZT+3TC	ND		34% (32)	
[33]	>10% loss of LF	NFV+(AZT+3TC or DDI+D4T)	NR			
		EFV+(AZT+3TC or DDI+D4T)	NR		ND	NA
			OR^c^ NFV vs EFV NR	0.06		

a. adjusted for NRTI arm, race, sex, age, baseline extremity fat and baseline CD4 count; b. if virologically suppressed for 3 months; c. adjusted for NRTI assignment, age, sex, race, and baseline BMI, HIV RNA and CD4 count.

LF: limb fat; NA: not applicable; ND: not done; NR: not reported; OR: odds ratio.

Antiretrovirals: 3TC: lamivudine; AZT: zidovudine; D4T: stavudine; DDI: didanosine; EFV: efavirenz; LPVr: ritonavir-boosted lopinavir; NFV: nelfinavir; TDF: tenofovir.

**Table 9:** Proportion of patients with peripheral lipoatrophy on DEXA scan: NRTI versus NRTI.

Study	Definition	Arm	Week 48 (n)	p value	Week 96 (n)	p value
[33]	>10% loss of LF	DDI+D4T+(EFV or NFV)	NR			
		AZT+3TC+(EFV or NFV)	NR		ND	NA
			OR^a^ DDI+D4T vs AZT+3TC 3.3. 95% CI 1.2 to 8.6	0.02		

a. adjusted for age, sex, race, and baseline BMI, HIV RNA and CD4 count.

LF: limb fat; NA: not applicable; ND: not done; NR: not reported; OR: odds ratio.

Antiretrovirals: 3TC: lamivudine; AZT: zidovudine; D4T: stavudine; DDI: didanosine; EFV: efavirenz; NFV: nelfinavir.

**Table 10:** Proportion of patients with peripheral lipoatrophy on DEXA scan: PI versus PI.

Study	Definition	Arm	Week 48 (n)	p value	Week 96 (n)	p value
[15]	≥20% loss of LF	ATVr+D4T +3TC	21% (72)	NR	29% (55)	<0.05
		ATV+D4T +3TC	30% (89)		49% (67)	

LF: limb fat; NR: not reported.

Antiretrovirals: 3TC: lamivudine; ATV: atazanavir; ATVr: ritonavir-boosted atazanavir; D4T: stavudine.

**Table 11:** Proportion of patients with peripheral lipoatrophy on DEXA scan: raltegravir versus efavirenz.

Study	Definition	Arm	Week 48 (n)	p value	Week 96 (n)	p value
[10]	≥20% loss of LF	RAL+TDF +FTC	ND	NA	8% (37)	0.62^a^
		EFV+TDF +FTC	ND		5% (38)	

a. p value calculated by the authors of this review.

LF: limb fat; NA: not applicable; ND: not done.

Antiretrovirals: EFV: efavirenz; FTC: emtricitabine; RAL: raltegravir; TDF: tenofovir.

In general, participants who received NRTI-sparing regimens gained more (or lost less) SAT or limb fat over time than those on NRTI-containing regimens. However one study that compared an abacavir-containing regimen with an NRTI-sparing regimen found no significant between-arm differences in change in subcutaneous thigh fat or SAT [32]. Participants who received PI-containing regimens also gained more (or lost less) SAT or limb fat over time than those on efavirenz-based regimens [3], [13], [19], [24], [33]. The incidence of peripheral lipoatrophy (defined as ≥20% loss of limb fat) was significantly lower in participants on NRTI-sparing regimens compared with those on NRTI-containing regimens, and in those on PI-containing regimens compared with those on EFV-based regimens [9], [11], [13], [19].

There were no significant differences in average gains in SAT or limb fat over time in the four studies that compared abacavir- with tenofovir-based regimens [3], [14], [16], [23]. One study found that participants who received an abacavir-containing regimen gained limb fat over time, in contrast to participants who received a stavudine-containing regimen who lost limb fat over time (gain of 913 g versus loss of 1578 g, p<0.001) [22]. Another study found that participants who received a zidovudine-lamivudine-containing regimen gained limb fat over time, in contrast to participants who received a stavudine-didanosine-containing regimen who lost limb fat over time (gain of 1.7% versus loss of 26.4%, p value not reported) [33].

Unboosted atazanavir was associated with significant reduction in limb fat at 96 weeks, while there was no significant change in limb fat in the ritonavir-boosted atazanavir arm (both arms were on stavudine and lamivudine) [15]. The proportion of participants with ≥20% loss in limb fat was significantly greater in the unboosted atazanavir arm at 96 weeks [15]. However, there were no significant between-group differences in absolute change from baseline in SAT or limb fat with ritonavir-boosted versus unboosted atazanavir [15]. There were no significant between-group differences in absolute change from baseline in SAT or limb fat in a study comparing ritonavir-boosted tipranavir (at the registered dose) with ritonavir-boosted lopinavir [17].

In the one study that compared raltegravir with efavirenz, average gains in limb fat over time and the incidence of lipoatrophy were similar in both groups [10].

#### Fat gain: changes in trunk fat or VAT with different antiretroviral regimens

In general, participants randomised to different ART regimens gained similar amounts of trunk fat or VAT over time. The changes from baseline in trunk fat and VAT are summarised in Tables 12, 13, 14, 15, 16.

**Table 12:** Change from baseline in trunk fat (TF) on DEXA scan, and visceral adipose tissue (VAT) on CT scan: NRTI-containing regimens versus NRTI-sparing regimens.

Study	Measure	Arm	Week 48 (n)	p value	Week 96 (n)	p value
[9]	TF^a^	LPVr+AZT+3TC	−211 g (22)	0.665	346 g (5)	>0.05
		LPVr monotherapy	−579 g (41)		−859 g (8)	
[4]			Percentage of body fat, 0 & 48 wks:^b^			
	TF^a^	LPVr+ continue 2NRTIs	20.6; 22.6 (33^c^)	NR	ND	NA
		NVP+LPVr	22.5; 24.0 (33^c^)		ND	
[12]			Mean (95% CI) cm^2^, 0 & 48 wks:^e^		Mean (95% CI) cm^2^, 96 wks:^e^	
	VAT^d^	AZT+3TC+LPVr	100 (88 to 112); 104 (90 to 118) (22^c^)	NR	122 (108 to 135)	NR
		NVP+LPVr	100 (89 to 111); 109 (96 to 122) (26^c^)		111 (98 to 124)	
[32]	VAT^a^	ABC+continue other ARVs	−15% (43)	0.1	ND	NA
		LPVr+NVP	−4% (47)		ND	
[21]	TF^a^	EFV+2NRTIs^f^	133 g^g^ (25)	>0.05	−583 g^g^ (25)	>0.05
		LPVr+EFV	−170 g^g^ (22)		−206 g^g^ (22)	
[11]	TF^a^	DRVr+ continue 2NRTIs	5.9% (74)	>0.05	ND	NA
		DRVr monotherapy	7.6% (67)		ND	

a. median; b. absolute values at each time-point (change from baseline not reported); c. n at baseline (n at time-point not stated); d. mean; e. means (corrected for differences in baseline values) and 95% confidence intervals (calculated by the authors of this review) at each time-point (change from baseline not reported); f. DDI+3TC or DDI+AZT or AZT+3TC or D4T+3TC or DDI+D4T; g. values derived from graph.

NA: not applicable; ND: not done; NR: not reported.

Antiretrovirals: 3TC: lamivudine; ABC: abacavir; AZT: zidovudine; D4T: stavudine; DDI: didanosine; DRVr: ritonavir-boosted darunavir; EFV: efavirenz; LPVr: ritonavir-boosted lopinavir; NVP: nevirapine.

**Table 13:** Change from baseline in trunk fat (TF) on DEXA scan, and visceral adipose tissue (VAT) on CT scan: PI versus NNRTI.

Study	Measure	Arm	Week 48 (n)	p value	Week 96 (n)	p value
[3]	TF^a^	ATVr+(ABC+3TC or TDF+FTC)	26.1%^b^ (105)	NR	36.5% (94)	0.028
		EFV+(ABC+3TC or TDF+FTC)	20.4%^b^ (112)		21.1% (109)	
	VAT	ATVr+(ABC+3TC or TDF+FTC)	NR	NA	LR coefficient^c^ (ATVr vs EFV) 11.0 cm^2^	0.20
		EFV+(ABC+3TC or TDF+FTC)	NR		95% CI −5.9 to 27.9	
[19]	TF^d^	LPVr+AZT+3TC or LPVr^e^	6.9% (NR)	NR	13.8%^b^ (74)	>0.05
		EFV+AZT+3TC	15.2% (NR)		14.6%^b^ (32)	
[24]	VAT^a^	ATV+AZT+3TC	15.3 cm^2^ (62). 95% CI for difference: −10.4 to 12.6	NR	ND	NA
		EFV+AZT+3TC	14.1 cm^2^ (46)		ND	
[20], [33]	TF^d,f^	NFV+(AZT+3TC or DDI+D4T)	8.3%^b^ (23)	NR	−6.8%^b^ (11)	NR
		EFV+(AZT+3TC or DDI+D4T)	14.8%^b^ (26)		32.6%^b^ (16)	

a. mean; b. values derived from graph; c. adjusted for treatment allocation, sex, age, race, and baseline HIV RNA, CD4 count and BMI; d. median; e. LPVr monotherapy if virologically suppressed for 3 months; f. as treated analysis.

LR: linear regression; NA: not applicable; ND: not done; NR: not reported.

Antiretrovirals: 3TC: lamivudine; ATV: atazanavir; ATVr: ritonavir-boosted atazanavir; AZT: zidovudine; D4T: stavudine; DDI: didanosine; EFV: efavirenz; FTC: emtricitabine; LPVr: ritonavir-boosted lopinavir; NFV: nelfinavir; TDF: tenofovir.

**Table 14:** Change from baseline in trunk fat (TF) on DEXA scan, and visceral adipose tissue (VAT) on CT scan: NRTI versus NRTI.

Study	Measure	Arm	Week 48 (n)	p value	Week 96 (n)	p value
[3]	TF^a^	ABC+3TC+(ATVr or EFV)	24.9%^b^ (107)	NR	29.4%^b^ (102)	0.76
		TDF+FTC+(ATVr or EFV)	21.6%^b^ (110)		27.3%^b^ (101)	
	VAT	ABC+3TC+(ATVr or EFV)	ND	NA	LR coefficient^c^ (ABC+3TC vs TDF+FTC) −5.3 cm^2^	0.52
		TDF+FTC+(ATVr or EFV)	ND		95% CI −21.5 to 11.0	
[22]	TF^a^	ABC+3TC+EFV	ND	NA	1225 g (25)	0.58
		D4T+3TC+EFV	ND		996 g (32)	
[23]	TF^a^	Switch AZT/D4T to ABC+cont. other ARVs	618 g (44)	0.97	ND	NA
		Switch AZT/D4T to TDF+cont. other ARVs	607 g (49)		ND	
	VAT^a^	Switch AZT/D4T to ABC+cont. other ARVs	2 cm^2^ (44)	0.49	ND	NA
		Switch AZT/D4T to TDF+cont. other ARVs	6.8 cm^2^ (49)		ND	
[20], [33]	TF^d,e^	DDI+D4T+(EFV or NFV)	9.8%b (42)	NR	−0.7%^b^ (22)	NR
		AZT+3TC+(EFV or NFV)	9.1%b (39)		13.6%^b^ (24)	

a. mean; b. values derived from graph; c. adjusted for treatment allocation, sex, age, race, and baseline HIV RNA, CD4 count and BMI; d. median; e. as treated analysis.

LR: linear regression; NA: not applicable; ND: not done; NR: not reported.

Antiretrovirals: 3TC: lamivudine; ABC: abacavir; ATVr: ritonavir-boosted atazanavir; AZT: zidovudine; D4T: stavudine; DDI: didanosine; EFV: efavirenz; FTC: emtricitabine; NFV: nelfinavir; TDF: tenofovir.

**Table 15:** Change from baseline in trunk fat (TF) on DEXA scan, and visceral adipose tissue (VAT) on CT scan: PI versus PI.

Study	Measure	Arm	Week 48 (n)	p value	Week 96 (n)	p value
[15]	TF^a^	ATVr+D4T+3TC	12% (72)	NR	16% (55)	>0.05
		ATV+D4T+3TC	15% (89)		14% (67)	
	VAT^a^	ATVr+D4T+3TC	28% (68)	NR	33% (56)	>0.05
		ATV+D4T+3TC	34% (85)		32% (62)	
[17]	TF^b^	TPVr100+TDF+3TC	−0.8% (46)	0.005 vs LPVr+TDF+3TC	ND	NA
		TPVr200+TDF+3TC	−0.7% (48)	0.02 vs LPVr+TDF+3TC	ND	
		LPVr+TDF+3TC	2.1% (45)		ND	
	VAT^b^	TPVr100+TDF+3TC	−6 cm2 (46)	0.4 vs LPVr+TDF+3TC	ND	NA
		TPVr200+TDF+3TC	−9 cm2 (48)	0.04 vs LPVr+TDF+3TC	ND	
		LPVr+TDF+3TC	−3 cm2 (45)		ND	

a. mean; b. median.

NA: not applicable; ND: not done; NR: not reported.

Antiretrovirals: 3TC: lamivudine; ATV: atazanavir; ATVr: ritonavir-boosted atazanavir; D4T: stavudine; LPVr: ritonavir-boosted lopinavir; TDF: tenofovir; TPVr100: tipranavir/ritonavir 500/100 mg twice a day; TPVr200: tipranavir/ritonavir 500/200 mg twice a day.

**Table 16:** Change from baseline in trunk fat (TF) on DEXA scan, and visceral adipose tissue (VAT) on CT scan: raltegravir versus efavirenz.

Study	Measure	Arm	Week 48 (n)	p value	Week 96 (n)	p value
[10]	TF^a^	RAL+TDF +FTC	18.9% (40)	0.63^b^	21.6% (37)	0.71^b^
		EFV+TDF +FTC	22.6% (46)		25.5% (38)	

a. mean; b. p value calculated by authors of this review.

Antiretrovirals: EFV: efavirenz; FTC: emtricitabine; RAL: raltegravir; TDF: tenofovir.

There were no significant between-group differences in changes from baseline in trunk fat or VAT in the six studies that compared NRTI-sparing and NRTI-containing regimens [4], [9], [11], [12], [21], [32], and in the four studies that compared different NRTI-containing regimens [3], [22], [23], [33]. Similarly, there were no significant differences in the incidences of lipohypertrophy (defined as >20% gain in trunk fat) [9], [11], [19].

The results of studies that compared PIs and NNRTIs were not consistent. One study found that participants who received ritonavir-boosted atazanavir had significantly greater increases in trunk fat at week 96 than those who received efavirenz (36.5% versus 21.1% respectively, p = 0.028) [3]. Another study found no significant changes at week 48 in VAT between those who received unboosted atazanavir compared with those who received efavirenz (15.3 cm^2^ versus 14.1 cm^2^ respectively, 95% confidence interval for the difference: −10.4 to 12.6 cm^2^) [24]. There were no significant between-group differences in changes from baseline in trunk fat, or in incidence of lipohypertrophy in one study that compared ritonavir-boosted lopinavir and efavirenz [19]. One study found that participants who received efavirenz gained more trunk fat over time on average than those who received nelfinavir, however it was not reported whether or not the difference was statistically significant [33].

There were no significant between-group changes from baseline in trunk fat or VAT in those who received ritonavir-boosted atazanavir compared with unboosted atazanavir in one study [15]. A study that compared ritonavir-boosted tipranavir and ritonavir-boosted lopinavir found small, but statistically significant between-group differences in changes from baseline in both trunk fat and VAT: those who received ritonavir-boosted tipranavir lost trunk fat over time, while those who received ritonavir-boosted lopinavir gained trunk fat over time [17].

In the one study that compared raltegravir with efavirenz, gains in trunk fat over time were similar in both groups [10].

#### Changes in limb fat and SAT after switching antiretroviral regimens

In general, participants who were switched away from NRTI-containing, or more specifically thymidine analogue-containing, regimens gained limb fat over time, when compared with participants who continued NRTI- or thymidine analogue-containing regimens, who generally lost limb fat [27]–[30], [32], [34].

There were no significant between-group differences in changes from baseline in limb fat or SAT in studies that switched to NNRTI- from PI-containing regimens [31], to ritonavir-boosted atazanavir from other ritonavir-boosted PI regimens [25], or to raltegravir from PI regimens [26].

Changes from baseline in limb fat, subcutaneous thigh fat and SAT are summarized in Tables 17, 18, 19, 20, and 21.

**Table 17:** Switching studies: change from baseline in subcutaneous thigh fat (STF) and subcutaneous adipose tissue (SAT) on CT scan: NRTI-containing regimens versus NRTI-sparing regimens.

Study	Measure	Arm	Week 24 (n)	p value	Week 48 (n)	p value	Week 96 (n)	p value
[32]	STF^a^	Cont AZT or D4T regimen	−3% (24)	NR	ND	NA	ND	NA
		Switch to LPVr+NVP	8% (40)		ND		ND	
[29]	STF^b^	Cont NRTI regimen	ND	NA	0 cm^3^ (35)	0.004	11 cm^3^ (25)	0.001
		Switch to PI+NNRTI	ND		42 cm^3^ (41)		120 cm^3^ (28)	
	SAT^b^	Cont NRTI regimen	ND	NA	1 cm^3^ (35)	0.004	14 cm^3^ (23)	0.088
		Switch to PI+NNRTI	ND		22 cm^3^ (39)		31 cm^3^ (30)	

a. median; b. mean.

NA: not applicable; ND: not done; NR: not reported.

Antiretrovirals: AZT: zidovudine; D4T: stavudine; LPVr: ritonavir-boosted lopinavir; NVP: nevirapine.

**Table 18:** Switching studies: change from baseline in limb fat (LF) on DEXA scan: PI versus NNRTI.

Study	Measure	Arm	Week 24 (n)	p value	Week 48 (n)	p value	Week 96 (n)	p value
[31]						Mean (95% CI) 0 & 48 wks (kg):^d^		
	LF ^a,b^	Cont PI+ 2NRTIs	ND	NA	NR (54^c^)	1.5 (1.3 to 1.8); 1.3 (1.1 to 1.6)	ND	NA
		Switch to NVP+DDI+D4T	ND		NR (52^c^)	1.2 (1.1 to 1.4); 1.2 (1.1 to 1.4)	ND	

a. mean; b. leg fat; c. n at baseline (n at time-point not stated); d. means and 95% confidence intervals (calculated by the authors of this review) at each time-point (change from baseline not reported).

NA: not applicable; ND: not done; NR: not reported.

Antiretrovirals: D4T: stavudine; DDI: didanosine; NVP: nevirapine.

**Table 19:** Switching studies: change from baseline in limb fat (LF) on DEXA scan, and subcutaneous thigh fat (STF) and subcutaneous adipose tissue (SAT) on CT scan: NRTI versus NRTI.

Study	Measure	Arm	Week 24 (n)	p value	Week 48 (n)	p value	Week 96	p value
[28]	LF^a^	Cont AZT+3TC+EFV	ND	NA	−187 g (36). 95% CI for difference: 57 to 837 g	0.024	ND	NA
		Switch TDF+FTC+EFV	ND		261 g (38)		ND	
[32]	STF^b^	Cont AZT or D4T regimen	−3% (24)	NR	ND	NA	ND	NA
		Switch AZT/D4T to ABC	0% (37)		ND		ND	
[30]	LF^a^	Cont AZT or D4T regimen	0.08 kg (56)	0.02	ND	NA	ND	NA
		Switch AZT/D4T to ABC	0.39 kg (50)		ND		ND	
	STF^a,c^	Cont AZT or D4T regimen	−1.2 cm^2^ (56)	0.01	ND	NA	ND	NA
		Switch AZT/D4T to ABC	3.3 cm^2^ (50)		ND		ND	
	SAT^a^	Cont AZT or D4T regimen	−1.2 cm^2^ (56)	0.001	ND	NA	ND	NA
		Switch AZT/D4T to ABC	13.9 cm^2^ (50)		ND		ND	
[27]	SAT^b^	Cont AZT+3TC	−2.7%^d^(NR)	NR	−2.7%^d^ (59)	0.03	ND	NA
		Switch to TDF+FTC	2.1%^d^ (NR)		1.6%^d^ (66)		ND	
	LF^b^	Cont AZT+3TC	3.2%^d^ (NR)	NR	1.1%^d^ (59)	0.5	ND	NA
		Switch to TDF+FTC	3.9%^d^ (NR)		5.2%^d^ (66)		ND	

a. mean; b. median; c. right thigh; d. values derived from graph.

NA: not applicable; ND: not done; NR: not reported.

Antiretrovirals: 3TC: lamivudine; ABC: abacavir; AZT: zidovudine; D4T: stavudine; EFV: efavirenz; FTC: emtricitabine; TDF: tenofovir.

**Table 20:** Switching studies: change from baseline in limb fat (LF) on DEXA scan and subcutaneous adipose tissue (SAT) on CT scan: PI versus PI.

Study	Measure	Arm	Week 24 (n)	p value	Week 48 (n)	p value	Week 96 (n)	p value
[25]	LF^a^	Continue PIr +2NRTIs	ND	NA	−3.6% (54)	0.15	−6.1% (54)	0.17
		Switch PIr to ATVr	ND		0.9% (112)		−0.8% (112)	
	SAT^a^	Continue PIr +2NRTIs	ND	NA	−5.9% (59)	0.16	−9.7% (59)	0.6
		Switch PIr to ATVr	ND		−2.1% (108)		−3.5% (108)	

a. mean.

NA: not applicable; ND: not done.

Antiretrovirals: ATVr: ritonavir-boosted atazanavir; PIr: ritonavir-boosted PI.

**Table 21:** Switching studies: change from baseline in limb fat (LF) on DEXA scan and subcutaneous adipose tissue (SAT) on CT scan: PI versus raltegravir.

Study	Measure	Arm	Week 24 (n)	p value	Week 48 (n)	p value	Week 96 (n)	p value
[26]	LF^a^	Cont PI regimen	ND	NA	171 g (35)	0.791	ND	NA
		Switch PI to RAL	ND		32 g (39)		ND	
	SAT^a^	Cont PI regimen	ND	NA	3.6% (35)	0.496	ND	NA
		Switch PI to RAL	ND		−1.9% (39)		ND	

a. median.

NA: not applicable; ND: not done; RAL: raltegravir.

#### Changes in trunk fat or VAT after switching antiretroviral regimens

In general, participants who were switched away from NRTI-containing regimens, or from thymidine analogue-containing regimens, had similar increases in trunk fat over time to those who continued NRTI- or thymidine analogue-containing regimens [29], [30].

There were no significant between-group differences in changes from baseline in trunk fat or VAT in studies that switched to NNRTI- from PI-containing regimens [31], to ritonavir-boosted atazanavir from other ritonavir-boosted PI regimens [25], or to raltegravir from PI regimens [26].

Changes from baseline in trunk fat and VAT are summarised in Tables 22, 23, 24, 25, and 26.

**Table 22:** Switching studies: change from baseline in visceral adipose tissue (VAT) on CT scan: NRTI-containing versus NRTI-sparing regimens.

Study	Measure	Arm	Week 24 (n)	p value	Week 48 (n)	p value	Week 96 (n)	p value
[29]	VAT^a^	Cont NRTI regimen	ND	NA	5 cm^3^ (35)	0.987	17 cm^3^ (23)	0.566
		Switch to PI+NNRTI	ND		7 cm^3^ (39)		6 cm^3^ (30)	

a. mean.

NA: not applicable; ND: not done.

**Table 23:** Switching studies: change from baseline in trunk fat (TF) on DEXA scan: PI versus NNRTI.

Study	Measure	Arm	Week 24	p value	Week 48 (n)	p value	Week 96	p value
[31]						Mean (95% CI) 0 & 48 wks (kg):^c^		
	TF ^a^	Cont PI+ 2NRTIs	ND	NA	NR (54^b^)	7.8 (6.9 to 8.7); 8.0 (6.9 to 9.1)	ND	NA
		Switch to NVP +DDI+D4T	ND		NR (52^b^)	6.3 (5.7 to 6.9); 5.9 (5.2 to 6.7)	ND	

a. mean; b. n at baseline (n at time-point not stated); c. means and 95% confidence intervals (calculated by the authors of this review) at each time-point (change from baseline not reported).

NA: not applicable; ND: not done; NR: not reported.

Antiretrovirals: D4T: stavudine; DDI: didanosine; NVP: nevirapine.

**Table 24:** Switching studies: change from baseline in trunk fat (TF) on DEXA scan and visceral adipose tissue (VAT) on CT scan: NRTI versus NRTI.

Study	Measure	Arm	Week 24 (n)	p value	Week 48 (n)	p value	Week 96 (n)	p value
[28]	TF^a^	Cont AZT+ 3TC+EFV	ND	NA	358 g	>0.05	ND	NA
		Switch TDF+ FTC+EFV	ND		130 g		ND	
[30]	TF^a^	Cont AZT or D4T regimen	0.8 kg (56)	0.31	ND	NA	ND	NA
		Switch AZT/ D4T to ABC	1.4 kg (50)		ND		ND	
	VAT^a^	Cont AZT or D4T regimen	−1.3 cm^2^ (56)	0.07	ND	NA	ND	NA
		Switch AZT/ D4T to ABC	1.2 cm^2^ (50)		ND		ND	

a. mean.

NA: not applicable; ND: not done.

Antiretrovirals: 3TC: lamivudine; ABC: abacavir; AZT: zidovudine; D4T: stavudine; DDI: didanosine; EFV: efavirenz; TDF: tenofovir.

**Table 25:** Switching studies: change from baseline in trunk fat (TF) on DEXA scan and visceral adipose tissue (VAT) on CT scan: PI versus PI.

Study	Measure	Arm	Week 24 (n)	p value	Week 48 (n)	p value	Week 96 (n)	p value
[25]	TF^a^	Continue PIr +2NRTIs	ND	NA	−1.8% (57)	0.14	−3.6% (57)	0.14
		Switch PIr to ATVr	ND		2.6% (112)		1.6% (112)	
	VAT^a^	Continue PIr +2NRTIs	ND	NA	−0.5% (59)	0.27	1.6% (59)	0.68
		Switch PIr to ATVr	ND		4.6% (108)		3.4% (108)	

a. mean.

NA: not applicable; ND: not done.

Antiretrovirals: ATVr: ritonavir-boosted atazanavir; PIr: ritonavir-boosted PI.

**Table 26:** Switching studies: change from baseline in trunk fat (TF) on DEXA scan and visceral adipose tissue (VAT) on CT scan: PI versus raltegravir.

Study	Measure	Arm	Week 24 (n)	p value	Week 48 (n)	p value	Week 96 (n)	p value
[26]	TF^a^	Cont PI regimen	ND	NA	382 g (35)	0.729	ND	NA
		Switch PI to RAL	ND		−28 g (39)		ND	
	VAT^a^	Cont PI regimen	ND	NA	11.9% (35)	0.936	ND	NA
		Switch PI to RAL	ND		12.8% (39)		ND	

a. median.

NA: not applicable; ND: not done; RAL: raltegravir.

## Discussion

We found overwhelming evidence that lipoatrophy is an antiretroviral adverse drug reaction. Subcutaneous fat volumes are considerably lower in patients on ART than in controls, subcutaneous fat loss progresses on ART, is associated with stavudine and zidovudine use, and partially reverses after switching to abacavir, tenofovir or an NRTI-sparing regimen. By contrast, central fat gain does not appear to be an antiretroviral adverse drug reaction. Visceral and trunk fat volume is no different in women on ART compared with control women, and is less in men on ART than in control men. Visceral fat accumulates at the same rate in patients on ART and controls. Finally, central fat gain generally occurs at similar rates in HIV-infected patients randomised to different ART regimens, is not associated with any specific antiretroviral drug or drug class, and does not reverse on switching antiretrovirals. We believe that this evidence indicates that central fat gain is a consequence of treating HIV infection, which normalizes the concentrations of inflammatory markers such as TNF-α (tumour necrosis factor alpha) that are known to cause wasting [36]. Lipoatrophy occurring together with central fat gain results in an unusual appearance, which may have persuaded clinicians that the fat gain is an antiretroviral adverse drug reaction. The fact that diet and exercise have been shown to improve central fat gain in patients on antiretroviral therapy provides some support for our conclusion that it is a consequence of lifestyle [37]–[39].

Efavirenz is associated with a higher risk of limb fat loss than PIs when combined with NRTIs that cause fat loss. A possible explanation for this observation is that the anti-apoptotic properties of PIs partially ameliorate the loss of adipocytes by increased apoptosis that is induced by NRTIs [40]. The observation that unboosted atazanavir, which is a non-peptidomimetic PI that does not have anti-apoptotic properties, is associated with more limb fat loss than ritonavir (a peptidomimetic PI)-boosted atazanavir supports this hypothesis. Alternatively, efavirenz may increase the adipocyte toxicity of thymidine analogue NRTIs. Efavirenz has been shown to be more toxic to adipocytes and to release more inflammatory cytokines than lopinavir-ritonavir [41]. Furthermore, efavirenz also displays mitochondrial toxicity in hepatocytes [42]; although we could find no data to support this, it is possible that it has a similar effect on adipocytes. Mitochondrial toxicity is thought to be a key mechanism of thymidine analogue-induced lipoatrophy [3].

Our study has several limitations. First, many of the studies that reported objective measures of fat redistribution by ART regimen were convenience sub-studies of randomised controlled trials, therefore patients undergoing DEXA and/or CT scans were not randomised. However, fat distribution sub-studies generally reported that the characteristics of the patients undergoing imaging of fat tissue were not different from the parent trial population. Many RCTs were powered according to efficacy end-points, and not for objective measures of fat distribution. Second, many studies reported only summary statistics and different outcome measures were reported, which prevented us from pooling data from different studies. Third, some studies did not report statistical analyses of changes in body fat distribution by study arm. Fourth, we were unable to assess the role of older protease inhibitors (other than nelfinavir) or nevirapine in fat redistribution as no studies with those antiretrovirals fulfilled our inclusion criteria. Fifth, we found only one study that compared fat changes over time in patients on ART and HIV-infected controls that was conducted in the United States. This limits our ability to generalise findings to other populations. Finally, we cannot exclude the causative role of specific antiretrovirals in focal forms of fat gain, such as buffalo humps, as the included studies reported trunk or visceral fat changes only.

In conclusion, our systematic review supports the hypothesis that peripheral lipoatrophy, but not central fat gain, is an antiretroviral adverse drug reaction. Lipoatrophy can be avoided and at least partially reversed by avoiding thymidine analogue nucleoside reverse transcriptase inhibitors. Central fat gain appears to be a consequence of treating HIV-infection, and reflects patterns of fat gain seen in the HIV-uninfected population.

## Supporting Information

Table S1 **Excluded studies.**
(DOCX)

Table S2 **Risk of bias.**
(DOCX)
