# Supplementary material for: Systematic Review of Antiretroviral-Associated Lipodystrophy: Lipoatrophy, but Not Central Fat Gain, Is an Antiretroviral Adverse Drug Reaction
Source: PLoS One. 2013 May 28;8(5):e63623. doi: 10.1371/journal.pone.0063623 (PMC3665842; doi:10.1371/journal.pone.0063623)
Supplement: Table S1 — Excluded studies. (DOCX) [file pone.0063623.s001.docx]

**Supplementary table S1: Excluded studies**

| **Excluded studies** | **Reason for exclusion** |
| --- | --- |
| [[1-9](#_ENREF_1)] | Not randomised |
| [[10-14](#_ENREF_10)] | Study not in adults |
| [[15-25](#_ENREF_15)] | No MRI, CT or DEXA measures done or reported |
| [[26-30](#_ENREF_26)] | No repeated measures done or reported |
| [[31-34](#_ENREF_31)] | No baseline measures done or reported |
| [[35](#_ENREF_35)] | DEXA results not presented by study arm |
| [[36](#_ENREF_36), [37](#_ENREF_37)] | No control group (cohort studies) |
| [[38-46](#_ENREF_38)] | Fewer than 20 participants in each arm |
| [[47](#_ENREF_47)] | Regimen included hydroxyurea |
| [[48](#_ENREF_48)] | Comparison was treatment strategy, not drug regimen |
| [[49](#_ENREF_49)] | Compared two different formulations of the same drug |

## References

1. Ferrer E, del Rio L, Martinez E, et al. Impact of switching from lopinavir/ritonavir to atazanavir/ritonavir on body fat redistribution in virologically suppressed HIV-infected adults. AIDS research and human retroviruses **2011** Oct;27(10):1061-5.

2. Madruga JR, Cassetti I, Suleiman JM, et al. The safety and efficacy of switching stavudine to tenofovir df in combination with lamivudine and efavirenz in hiv-1-infected patients: three-year follow-up after switching therapy. HIV clinical trials **2007** Nov-Dec;8(6):381-90.

3. Cassetti I, Madruga JV, Suleiman JM, et al. The safety and efficacy of tenofovir DF in combination with lamivudine and efavirenz through 6 years in antiretroviral-naive HIV-1-infected patients. HIV clinical trials **2007** May-Jun;8(3):164-72.

4. Dzwonek AB, Lawson MS, Cole TJ, Novelli V. Body fat changes and lipodystrophy in HIV-infected children: impact of highly active antiretroviral therapy. J Acquir Immune Defic Syndr **2006** Sep;43(1):121-3.

5. Martin A, Smith DE, Carr A, et al. Reversibility of lipoatrophy in HIV-infected patients 2 years after switching from a thymidine analogue to abacavir: the MITOX Extension Study. AIDS **2004** Apr 30;18(7):1029-36.

6. Martin A, Smith D, Carr A, et al. Progression of lipodystrophy (LD) with continued thymidine analogue usage: long-term follow-up from a randomized clinical trial (the PIILR study). HIV clinical trials **2004** Jul-Aug;5(4):192-200.

7. McComsey G, Lonergan T, Fisher R, et al. Improvements in lipoatrophy are observed after 24 weeks when stavudine (d4T) is replaced by either abacavir (ABC) or zidovudine (ZDV). Conference on Retroviruses and Opportunistic Infections. Seattle, **2002**.

8. Tashima K, Morales-Ramirez J, Butcher D, et al. Impact on Fat Loss of ZDV + 3TC Backbone: Abdominal CT-scan Substudy of Study DMP 266-006. Conference on Retroviruses and Opportunistic Infections. Boston, **2003**.

9. McComsey GA, Paulsen DM, Lonergan JT, et al. Improvements in lipoatrophy, mitochondrial DNA levels and fat apoptosis after replacing stavudine with abacavir or zidovudine. AIDS **2005**;19(1):15-23.

10. Spoulou V, Kanaka-Gantenbein C, Bathrellou I, et al. Monitoring of lipodystrophic and metabolic abnormalities in HIV-1 infected children on antiretroviral therapy. Hormones **2011** Apr-Jun;10(2):149-55.

11. Chantry CJ, Cervia JS, Hughes MD, et al. Predictors of growth and body composition in HIV-infected children beginning or changing antiretroviral therapy. HIV medicine **2010** Oct 1;11(9):573-83.

12. Arpadi SM, Bethel J, Horlick M, et al. Longitudinal changes in regional fat content in HIV-infected children and adolescents. AIDS **2009** Jul 31;23(12):1501-9.

13. Vigano A, Brambilla P, Cafarelli L, et al. Normalization of fat accrual in lipoatrophic, HIV-infected children switched from stavudine to tenofovir and from protease inhibitor to efavirenz. Antiviral therapy **2007**;12(3):297-302.

14. Vigano A, Mora S, Testolin C, et al. Increased lipodystrophy is associated with increased exposure to highly active antiretroviral therapy in HIV-infected children. J Acquir Immune Defic Syndr **2003** Apr 15;32(5):482-9.

15. Bussmann H, Wester CW, Thomas A, et al. Response to zidovudine/didanosine-containing combination antiretroviral therapy among HIV-1 subtype C-infected adults in Botswana: two-year outcomes from a randomized clinical trial. J Acquir Immune Defic Syndr **2009** May 1;51(1):37-46.

16. Jerico C, Knobel H, Montero M, et al. Hypertension in HIV-infected patients: prevalence and related factors. American journal of hypertension **2005** Nov;18(11):1396-401.

17. Sanchez-Conde M, de Mendoza C, Jimenez-Nacher I, Barreiro P, Gonzalez-Lahoz J, Soriano V. Reductions in stavudine dose might ameliorate mitochondrial-associated complications without compromising antiviral activity. HIV clinical trials **2005** Jul-Aug;6(4):197-202.

18. Negredo E, Paredes R, Bonjoch A, et al. Benefit of switching from a protease inhibitor (PI) to nevirapine in PI-experienced patients suffering acquired HIV-related lipodystrophy syndrome (AHL): interim analysis at 3 months of follow-up. Antiviral therapy **1999**;4 Suppl 3:23-8.

19. Joly V, Flandre P, Meiffredy V, et al. Increased risk of lipoatrophy under stavudine in HIV-1-infected patients: results of a substudy from a comparative trial. AIDS **2002** Dec 6;16(18):2447-54.

20. Brown TT, Chu H, Wang Z, et al. Longitudinal increases in waist circumference are associated with HIV-serostatus, independent of antiretroviral therapy. AIDS **2007**;21(13):1731-8.

21. Palella FJ, Cole SR, Chmiel JS, et al. Anthropometrics and Examiner-Reported Body Habitus Abnormalities in the Multicenter AIDS Cohort Study. Clinical infectious diseases : an official publication of the Infectious Diseases Society of America **2004** 15 March;38:903-7.

22. Shlay JC, Bartsch G, Peng G, et al. Long-term body composition and metabolic changes in antiretroviral naive persons randomized to protease inhibitor-, nonnucleoside reverse transcriptase inhibitor-, or protease inhibitor plus nonnucleoside reverse transcriptase inhibitor-based strategy. J Acquir Immune Defic Syndr **2007** Apr 15;44(5):506-17.

23. Ribera E, Paradineiro JC, Domingo P, et al. A randomized study comparing the efficacy and tolerability of low-dose versus standard dose stavudine in antiretroviral-naive patients (ETOX study). IAS Conference on HIV Pathogenesis and Treatment. Rio de Janeiro, **2005**.

24. Fisac C, Virgili N, Ferrer E, et al. A Comparison of the Effects of Nevirapine and Nelfinavir on Metabolism and Body Habitus in Antiretroviral-Naive Human Immunodeficiency Virus-Infected Patients: A Randomized Controlled Study. J Clin Endocrinol Metab **2003** November;88(11):5186-92.

25. Tien PC, Barron Y, Justman JE, et al. Antiretroviral therapies associated with lipoatrophy in HIV-infected women. AIDS patient care and STDs **2007** May;21(5):297-305.

26. Brown TT, Xu X, John M, et al. Fat distribution and longitudinal anthropometric changes in HIV-infected men with and without clinical evidence of lipodystrophy and HIV-uninfected controls: a substudy of the Multicenter AIDS Cohort Study. AIDS research and therapy **2009**;6:8.

27. Coloma Conde AG, Alvarez Albarran M, Roca-Cusachs Coll A, Domingo Pedrol P, Puig Campmany M. Prevalencia de hipertension arterial y perfil lipidico en pacientes con infeccion por el virus de la inmunodeficiencia humana. Med Clin (Barc) **2008**;131(18):681-4.

28. Smith B, Raper J, Weaver M, et al. Body composition, visceral fat and bone mineral density: A comparison of HIV infected and non-infected women. International AIDS Conference. Barcelona, **2002**.

29. van der Valk M, Casula M, Weverling G-J, et al. Prevalence of lipoatrophy and mitochondrial DNA content of blood and subcutaneous fat in HIV-1-infected patients randomly alocated to zidovudine- or stavudine-based therapy. Antiviral therapy **2004**;9:385-93.

30. Gallant J, DeJesus E, Arribas JR, et al. Tenofovir DF, Emtricitabine, and Efavirenz vs Zidovudine, Lamivudine, and Efavirenz for HIV. N Engl J Med **2006** January 19;354(3):251-60.

31. Arribas JR, Pozniak AL, Gallant JE, et al. Tenofovir disoproxil fumarate, emtricitabine, and efavirenz compared with zidovudine/lamivudine and efavirenz in treatment-naive patients: 144-week analysis. J Acquir Immune Defic Syndr **2008** Jan 1;47(1):74-8.

32. Pozniak AL, Gallant JE, DeJesus E, et al. Tenofovir disoproxil fumarate, emtricitabine, and efavirenz versus fixed-dose zidovudine/lamivudine and efavirenz in antiretroviral-naive patients: virologic, immunologic, and morphologic changes--a 96-week analysis. J Acquir Immune Defic Syndr **2006** Dec 15;43(5):535-40.

33. Gallant JE, Staszewski S, Pozniak AL, et al. Efficacy and Safety of Tenofovir DF vs Stavudine in Combination Therapy in Antiretroviral-Naive Patients. A 3-Year Randomized Trial. JAMA : the journal of the American Medical Association **2004**;292(2):191-201.

34. Murphy R, Katlama C, Weverling G-J, et al. Fat Redistribution and Metabolic Study (FRAMS) in Patients Receiving a Nucleoside REverse TRanscriptase Inhibitor, non-NRTI, or Protease Inhibitor-based REgimen over 4 Years: FRAMS 11 Substudy of the Atlantic Study. Conference on Retroviruses and Opportunistic Infections. San Francisco, **2004**.

35. Fisac C, Fumero E, Crespo M, et al. Metabolic benefits 24 months after replacing a protease inhibitor with abacavir, efavirenz or nevirapine. AIDS **2005**;19(9):917-25.

36. McDermott AY, Terrin N, Wanke C, Skinner S, Tchetgen E, Shevitz AH. CD4+ cell count, viral load, and highly active antiretroviral therapy use are independent predictors of body composition alterations in HIV-infected adults: a longitudinal study. Clinical infectious diseases : an official publication of the Infectious Diseases Society of America **2005** Dec 1;41(11):1662-70.

37. Pernerstorfer-Schoen H, Schindler K, Parschalk B, et al. Beneficial effects of protease inhibitors on body composition and energy expenditure: a comparison between HIV-infected and AIDS patients. AIDS **1999** Dec 3;13(17):2389-96.

38. Cooper DA, Cordery DV, Reiss P, et al. The effects of enfuvirtide therapy on body composition and metabolic parameters over 48 weeks in the TORO body imaging substudy. HIV medicine **2011** Jan;12(1):31-9.

39. Boothby M, McGee KC, Tomlinson JW, et al. Adipocyte differentiation, mitochondrial gene expression and fat distribution: differences between zidovudine and tenofovir after 6 months. Antiviral therapy **2009**;14(8):1089-100.

40. Lowe SH, Hassink EA, van Eck-Smit BL, Borleffs JC, Lange JM, Reiss P. Stavudine but not didanosine as part of HAART contributes to peripheral lipoatrophy: a substudy from the Antiretroviral Regimen Evaluation Study (ARES). HIV clinical trials **2007** Sep-Oct;8(5):337-44.

41. Cameron DW, Becker S, King MS, et al. Exploratory study comparing the metabolic toxicities of a lopinavir/ritonavir plus saquinavir dual protease inhibitor regimen versus a lopinavir/ritonavir plus zidovudine/lamivudine nucleoside regimen. The Journal of antimicrobial chemotherapy **2007** May;59(5):957-63.

42. Moyle GJ, Baldwin C, Langroudi B, Mandalia S, Gazzard BG. A 48-week, randomized, open-label comparison of three abacavir-based substitution approaches in the management of dyslipidemia and peripheral lipoatrophy. J Acquir Immune Defic Syndr **2003** May 1;33(1):22-8.

43. Stanley TL, Joy T, Hadigan CM, et al. Effects of switching from lopinavir/ritonavir to atazanavir/ritonavir on muscle glucose uptake and visceral fat in HIV-infected patients. AIDS **2009** Jul 17;23(11):1349-57.

44. Milinkovic A, Martinez E, Lopez S, et al. The impact of reducing stavudine dose versus switching to tenofovir on plasma lipids, body composition and mitochondrial function in HIV-infected patients. Antiviral therapy **2007**;12(3):407-15.

45. John M, McKinnon EJ, James IR, et al. Randomized, controlled, 48-week study of switching stavudine and/or protease inhibitors to combivir/abacavir to prevent or reverse lipoatrophy in HIV-infected patients. J Acquir Immune Defic Syndr **2003** May 1;33(1):29-33.

46. Lake J, McComsey GA, Hulgan T, et al. Raltegravir as replacement for PI- or NNRTI-based ART in HIV-infected women with lipohypertrophy: the women, integrase, and fat accumulation trial. IAS Conference on HIV Pathogenesis, Treatment and Prevention. Rome, **2011**.

47. Carr A, Hudson J, Chuah J, et al. HIV protease inhibitor substitution in patients with lipodystrophy: a randomized, controlled, open-label, multicentre study. AIDS **2001** Sep 28;15(14):1811-22.

48. Martinez E, Visnegarwala F, Grund B, et al. The effects of intermittent, CD4-guided antiretroviral therapy on body composition and metabolic parameters. AIDS **2010** Jan 28;24(3):353-63.

49. Yang Y, Wilder-Smith A, Panchalingam A, Tha NO, Paton NI. Changes in body fat measured by DEXA in patients taking different formulations of stavudine. HIV clinical trials **2005** Nov-Dec;6(6):337-43.
