# Supplementary material for: Systematic Review of Antiretroviral-Associated Lipodystrophy: Lipoatrophy, but Not Central Fat Gain, Is an Antiretroviral Adverse Drug Reaction
Source: PLoS One. 2013 May 28;8(5):e63623. doi: 10.1371/journal.pone.0063623 (PMC3665842; doi:10.1371/journal.pone.0063623)
Supplement: Table S2 — Risk of bias. (DOCX) [file pone.0063623.s002.docx]

**Supplementary table S2: risk of bias**

| **Study** | **Random sequence generation** | **Allocation concealment** | **Blinding of participants and personnel** | **Blinding of outcome assessment** | **Incomplete outcome data** | **Selective outcome reporting** | **Other bias** | **Reason for other risk of bias** |
| --- | --- | --- | --- | --- | --- | --- | --- | --- |
| [[1](#_ENREF_1)] | NA | NA | NA | Unclear | Unclear | Unclear | High | a |
| [[2](#_ENREF_2)] | Unclear | Unclear | High | Low | High | Low | Unclear | b |
| [[3](#_ENREF_3)] | Unclear | Unclear | High | Low | Unclear | Low | High | c |
| [[4](#_ENREF_4)] | Low | Low | Low | Unclear | High | Unclear | High | c |
| [[5](#_ENREF_5)] | Unclear | Unclear | High | Low | Low | Unclear | High | c |
| [[6](#_ENREF_6)] | Unclear | Unclear | High | Unclear | High | Low | Low |  |
| [[7](#_ENREF_7)] | Low | Low | Unclear | Low | Low | Unclear | Low |  |
| [[8](#_ENREF_8)] | Unclear | Unclear | High | Low | High | Low | Low |  |
| [[9](#_ENREF_9)] | High | Unclear | High | Low | Unclear | Unclear | High | c |
| [[10](#_ENREF_10)] | Unclear | Unclear | High | Unclear | Unclear | Unclear | Low |  |
| [[11](#_ENREF_11)] | Unclear | Unclear | High | Low | Low | Low | Low |  |
| [[12](#_ENREF_12)] | Low | Low | High | Unclear | Low | Unclear | Low |  |
| [[13](#_ENREF_13)] | Unclear | Unclear | High | Unclear | High | Unclear | High | c |
| [[14](#_ENREF_14)] | Low | Unclear | High | Unclear | High | High | Low |  |
| [[15](#_ENREF_15)] | Unclear | Unclear | High | Unclear | Unclear | Unclear | Low |  |
| [[16](#_ENREF_16), [17](#_ENREF_17)] | Low | Unclear | High | Low | Unclear | Unclear | High | c |
| [[18](#_ENREF_18)] | Low | Low | High | Unclear | High | Unclear | High | c |
| [[19](#_ENREF_19)] | Unclear | Unclear | High | Low | High | Unclear | High | c |
| [[20](#_ENREF_20)] | Low | Unclear | High | Unclear | High | Low | Low |  |
| [[21](#_ENREF_21)] | Unclear | Unclear | Low | Unclear | High | Unclear | High | c |
| [[22](#_ENREF_22)] | Low | Low | High | Low | Low | Low | Low |  |
| [[23](#_ENREF_23)] | Low | Unclear | High | Low | High | Unclear | High | c |
| [[24](#_ENREF_24)] | Unclear | Unclear | High | Unclear | Unclear | Low | Low |  |
| [[25](#_ENREF_25)] | Low | Unclear | High | Unclear | Low | Unclear | Low | d |
| [[26](#_ENREF_26)] | Low | Unclear | High | Low | High | Low | Low |  |
| [[27](#_ENREF_27)] | Unclear | Unclear | High | Low | Low | Unclear | Low |  |
| [[28](#_ENREF_28)] | Unclear | Unclear | High | Low | High | Unclear | Low |  |

a. Cohort study; b. Unclear if sub-study randomised separately; c. Sub-study of larger randomised trial; d. Sub-study had separate randomisation list. NA: not applicable.

## References

1. Grunfeld C, Saag M, Cofrancesco J, Jr., et al. Regional adipose tissue measured by MRI over 5 years in HIV-infected and control participants indicates persistence of HIV-associated lipoatrophy. AIDS **2010** Jul 17;24(11):1717-26.

2. McComsey GA, Kitch D, Sax PE, et al. Peripheral and central fat changes in subjects randomized to abacavir-lamivudine or tenofovir-emtricitabine with atazanavir-ritonavir or efavirenz: ACTG Study A5224s. Clinical infectious diseases : an official publication of the Infectious Diseases Society of America **2011** Jul 15;53(2):185-96.

3. Kolta S, Flandre P, Van PN, et al. Fat tissue distribution changes in HIV-infected patients treated with lopinavir/ritonavir. Results of the MONARK trial. Current HIV research **2011** Jan;9(1):31-9.

4. Lennox JL, Dejesus E, Berger DS, et al. Raltegravir versus Efavirenz regimens in treatment-naive HIV-1-infected patients: 96-week efficacy, durability, subgroup, safety, and metabolic analyses. J Acquir Immune Defic Syndr **2010** Sep;55(1):39-48.

5. Valantin MA, Flandre P, Kolta S, et al. Fat Tissue Distribution Changes in HIV-infected Patients with Viral Suppression Treated with Darunavir/ritonavir (DRV/r) monotherapy versus 2 NRTIs + DRV/r in the MONOI-ANRS 136 Randomized Trial: Results at 48 weeks. Conference on Retroviruses and Opportunistic Infections. San Francisco, **2010**.

6. Negredo E, Miro O, Rodriguez-Santiago B, et al. Improvement of mitochondrial toxicity in patients receiving a nucleoside reverse-transcriptase inhibitor-sparing strategy: results from the Multicenter Study with Nevirapine and Kaletra (MULTINEKA). Clinical infectious diseases : an official publication of the Infectious Diseases Society of America **2009** Sep 15;49(6):892-900.

7. van Vonderen MG, van Agtmael MA, Hassink EA, et al. Zidovudine/lamivudine for HIV-1 infection contributes to limb fat loss. PloS one **2009**;4(5):e5647.

8. Haubrich RH, Riddler SA, DiRienzo AG, et al. Metabolic outcomes in a randomized trial of nucleoside, nonnucleoside and protease inhibitor-sparing regimens for initial HIV treatment. AIDS **2009** Jun 1;23(9):1109-18.

9. Martinez E, Arranz JA, Podzamczer D, et al. A simplification trial switching from nucleoside reverse transcriptase inhibitors to once-daily fixed-dose abacavir/lamivudine or tenofovir/emtricitabine in HIV-1-infected patients with virological suppression. J Acquir Immune Defic Syndr **2009** Jul 1;51(3):290-7.

10. McComsey G, Rightmire A, Wirtz V, Yang R, Mathew M, McGrath D. Changes in body composition with ritonavir-boosted and unboosted atazanavir treatment in combination with Lamivudine and Stavudine: a 96-week randomized, controlled study. Clinical infectious diseases : an official publication of the Infectious Diseases Society of America **2009** May 1;48(9):1323-6.

11. Tebas P, Zhang J, Hafner R, et al. Peripheral and visceral fat changes following a treatment switch to a non-thymidine analogue or a nucleoside-sparing regimen in HIV-infected subjects with peripheral lipoatrophy: results of ACTG A5110. The Journal of antimicrobial chemotherapy **2009** May;63(5):998-1005.

12. Martin A, Bloch M, Amin J, et al. Simplification of antiretroviral therapy with tenofovir-emtricitabine or abacavir-Lamivudine: a randomized, 96-week trial. Clinical infectious diseases : an official publication of the Infectious Diseases Society of America **2009** Nov 15;49(10):1591-601.

13. Carr A, Ritzhaupt A, Zhang W, et al. Effects of boosted tipranavir and lopinavir on body composition, insulin sensitivity and adipocytokines in antiretroviral-naive adults. AIDS **2008** Nov 12;22(17):2313-21.

14. Duvivier C, Ghosn J, Assoumou L, et al. Initial therapy with nucleoside reverse transcriptase inhibitor-containing regimens is more effective than with regimens that spare them with no difference in short-term fat distribution: Hippocampe-ANRS 121 Trial. The Journal of antimicrobial chemotherapy **2008** Oct;62(4):797-808.

15. Cameron DW, da Silva BA, Arribas JR, et al. A 96-week comparison of lopinavir-ritonavir combination therapy followed by lopinavir-ritonavir monotherapy versus efavirenz combination therapy. The Journal of infectious diseases **2008** Jul 15;198(2):234-40.

16. Dube MP, Komarow L, Mulligan K, et al. Long-term body fat outcomes in antiretroviral-naive participants randomized to nelfinavir or efavirenz or both plus dual nucleosides. Dual X-ray absorptiometry results from A5005s, a substudy of Adult Clinical Trials Group 384. J Acquir Immune Defic Syndr **2007** Aug 15;45(5):508-14.

17. Dube MP, Parker RA, Tebas P, et al. Glucose metabolism, lipid, and body fat changes in antiretroviral-naive subjects randomized to nelfinavir or efavirenz plus dual nucleosides. AIDS **2005** Nov 4;19(16):1807-18.

18. Tebas P, Zhang J, Yarasheski K, et al. Switching to a protease inhibitor-containing, nucleoside-sparing regimen (lopinavir/ritonavir plus efavirenz) increases limb fat but raises serum lipid levels: results of a prospective randomized trial (AIDS clinical trial group 5125s). J Acquir Immune Defic Syndr **2007** Jun 1;45(2):193-200.

19. Podzamczer D, Ferrer E, Sanchez P, et al. Less lipoatrophy and better lipid profile with abacavir as compared to stavudine: 96-week results of a randomized study. J Acquir Immune Defic Syndr **2007** Feb 1;44(2):139-47.

20. Moyle GJ, Sabin CA, Cartledge J, et al. A randomized comparative trial of tenofovir DF or abacavir as replacement for a thymidine analogue in persons with lipoatrophy. AIDS **2006** Oct 24;20(16):2043-50.

21. Jemsek JG, Arathoon E, Arlotti M, et al. Body fat and other metabolic effects of atazanavir and efavirenz, each administered in combination with zidovudine plus lamivudine, in antiretroviral-naive HIV-infected patients. Clinical infectious diseases : an official publication of the Infectious Diseases Society of America **2006** Jan 15;42(2):273-80.

22. Moyle GJ, Andrade-Villanueva J, Girard PM, et al. A randomized comparative 96-week trial of boosted atazanavir versus continued boosted protease inhibitor in HIV-1 patients with abdominal adiposity. Antiviral therapy **2012**;17(4):689-700.

23. Curran A, Martinez E, Saumoy M, et al. Body composition changes after switching from protease inhibitors to raltegravir: SPIRAL-LIP substudy. AIDS **2012** Feb 20;26(4):475-81.

24. Feeney ER, Vrouenraets SME, Wit F, et al. Switching Zidovudine to Tenofovir Improves Subcutaneous Adipose Tissue Volume and mtDNA Content. Conference on Retroviruses and Opportunistic Infections. Boston, **2011**.

25. Fisher M, Moyle GJ, Shahmanesh M, et al. A randomized comparative trial of continued zidovudine/lamivudine or replacement with tenofovir disoproxil fumarate/emtricitabine in efavirenz-treated HIV-1-infected individuals. J Acquir Immune Defic Syndr **2009** Aug 15;51(5):562-8.

26. Valantin MA, Lanoy E, Bentata M, et al. Recovery of fat following a switch to nucleoside reverse transcriptase inhibitor-sparing therapy in patients with lipoatrophy: results from the 96-week randomized ANRS 108 NoNuke Trial. HIV medicine **2008** Oct;9(8):625-35.

27. Carr A, Workman C, Smith DE, et al. Abacavir substitution for nucleoside analogs in patients with HIV lipoatrophy: a randomized trial. JAMA : the journal of the American Medical Association **2002** Jul 10;288(2):207-15.

28. Ruiz L, Negredo E, Domingo P, et al. Antiretroviral treatment simplification with nevirapine in protease inhibitor-experienced patients with hiv-associated lipodystrophy: 1-year prospective follow-up of a multicenter, randomized, controlled study. J Acquir Immune Defic Syndr **2001** Jul 1;27(3):229-36.
